# Engineered FGF19^ΔKLB^ protects against intrahepatic cholestatic liver injury in ANIT-induced and Mdr2-/- mice model

**DOI:** 10.1186/s12896-023-00810-9

**Published:** 2023-10-03

**Authors:** Lu Shi, Tiantian Zhao, Lei Huang, Xiaomin Pan, Tianzhen Wu, Xin Feng, Taoli Chen, Jiamin Wu, Jianlou Niu

**Affiliations:** 1 School of Pharmaceutical Science, Wenzhou Medical University, Wenzhou, 325035 Zhejiang China; 2 Pingyang Affiliated Hospital of Wenzhou Medical University, Wenzhou, 325499 Zhejiang China

**Keywords:** FGF19, Hepatocellular carcinoma, Bile acids, Intrahepatic cholestasis, Inflammation

## Abstract

**Background:**

The major safety concern of the clinical application of wild type FGF19 (FGF19^WT^) emerges given that its extended treatment causes hepatocellular carcinoma. Therefore, we previously generated a safer FGF19 variant - FGF19^ΔKLB^, which have same effects on glycemic control and bile acid production but much less mitogenic activity. However, it remains unclear as to whether FGF19^ΔKLB^ ameliorates intrahepatic cholestasis.

**Results:**

We found that, similar to that of FGF19^WT^, the chronic administration of FGF19^ΔKLB^ protects mice from cholestatic liver injury in these two models. The therapeutic benefits of FGF19^ΔKLB^ on cholestatic liver damage are attributable, according to the following mechanistic investigation, to the reduction of BA production, liver inflammation, and fibrosis. More importantly, FGF19^ΔKLB^ did not induce any tumorigenesis effects during its prolonged treatment.

**Conclusions:**

Together, our findings raise hope that FGF19^ΔKLB^ may represent a useful therapeutic strategy for the treatment of intrahepatic cholestasis.

**Supplementary Information:**

The online version contains supplementary material available at 10.1186/s12896-023-00810-9.

## Introduction

The liver synthesizes bile acids (BAs) from cholesterol, and the gallbladder stores them until they are released into the small intestine after eating. Intestinal nutrition absorption and the release of lipids, toxic compounds, and foreign organisms from the bile are two of BAs’ main roles [1]. Additionally, BAs are metabolic regulators that trigger nuclear receptors and G-protein-coupled receptors (GPCR) to maintain metabolic homeostasis by controlling levels of lipid and glucose [2]. Various disorders, including non-alcoholic fatty liver disease (NAFLD), diabetes, and inflammatory bowel disease, may result from disturbance of the intricate feedback and feedforward process that closely regulates the homeostasis of BAs [3].

After being reabsorbed in the ileum, BAs are transported back to the liver through enterohepatic circulation and then portal circulation, which is a key feedback mechanism to maintain their homeostasis [4]. Reportedly, the hepatic farnesoid X receptor (FXR)-small heterodimer partner (SHP) axis is crucial for this kind of negative feedback control [5]. Defects in the regulation of FXR targeted genes may impair the enterohepatic circulation of BAs and further lead to cholestatic liver disease. In these genes, the rate-limiting enzyme in the traditional route for the production of BAs is cholesterol 7 alpha-hydroxylase (Cyp7a1), whose transcription is inhibited by FXR [6, 7].

In the ileum, BA-dependent FXR activation causes the release of the enterokine FGF19 (FGF15 in the mouse). To stabilize SHP and dampen Cyp7a1 production, FGF19 functions as an endocrine hormone by attaching to the fibroblast growth factor receptor 4 (FGFR4)/ β-Klotho complex, which prevents the synthesis of BA [7–10]. Recently, FGF19 has been identified as a promising therapeutic target for diabetes, cholestatic liver disease, non-alcoholic steatohepatitis (NASH), and abnormalities of BA metabolism as a result of its remarkable pharmacological capabilities [11–14]. However, there are significant safety concerns due to the fact that long-term treatment with wild type FGF19 (FGF19^WT^) increases hepatocellular carcinoma [15–17]. For this reason, there has been an intensive search for non-mitogenic FGF19 variants preserving its metabolic activity [18–26]. We recently generated a safer FGF19 variant - FGF19^ΔKLB^, which suffer a sharp decline in mitogenic activity but are same in terms of inducing glycemic control and controlling BA synthesis [27]. However, it remains unclear as to whether FGF19^ΔKLB^ ameliorates intrahepatic cholestasis.

In this study, we assessed the FGF19^ΔKLB^’s therapeutic potential in ANIT-induced and *Mdr2*^−/−^ mice intrahepatic cholestasis models. We found that, similar to that of FGF19^WT^, administration of FGF19^ΔKLB^ improved hepatic functions and exerted anti-inflammation and anti-fibrosis effects in these models. More importantly, chronic treatment of FGF19^ΔKLB^ has little tumorigenesis effects, indicating that it could function as a viable treatment for associated intrahepatic cholestasis.

## Results

### FGF19^ΔKLB^ retains the activity of FGF19^WT^ in regulating BA metabolism

**Fig. 1:**
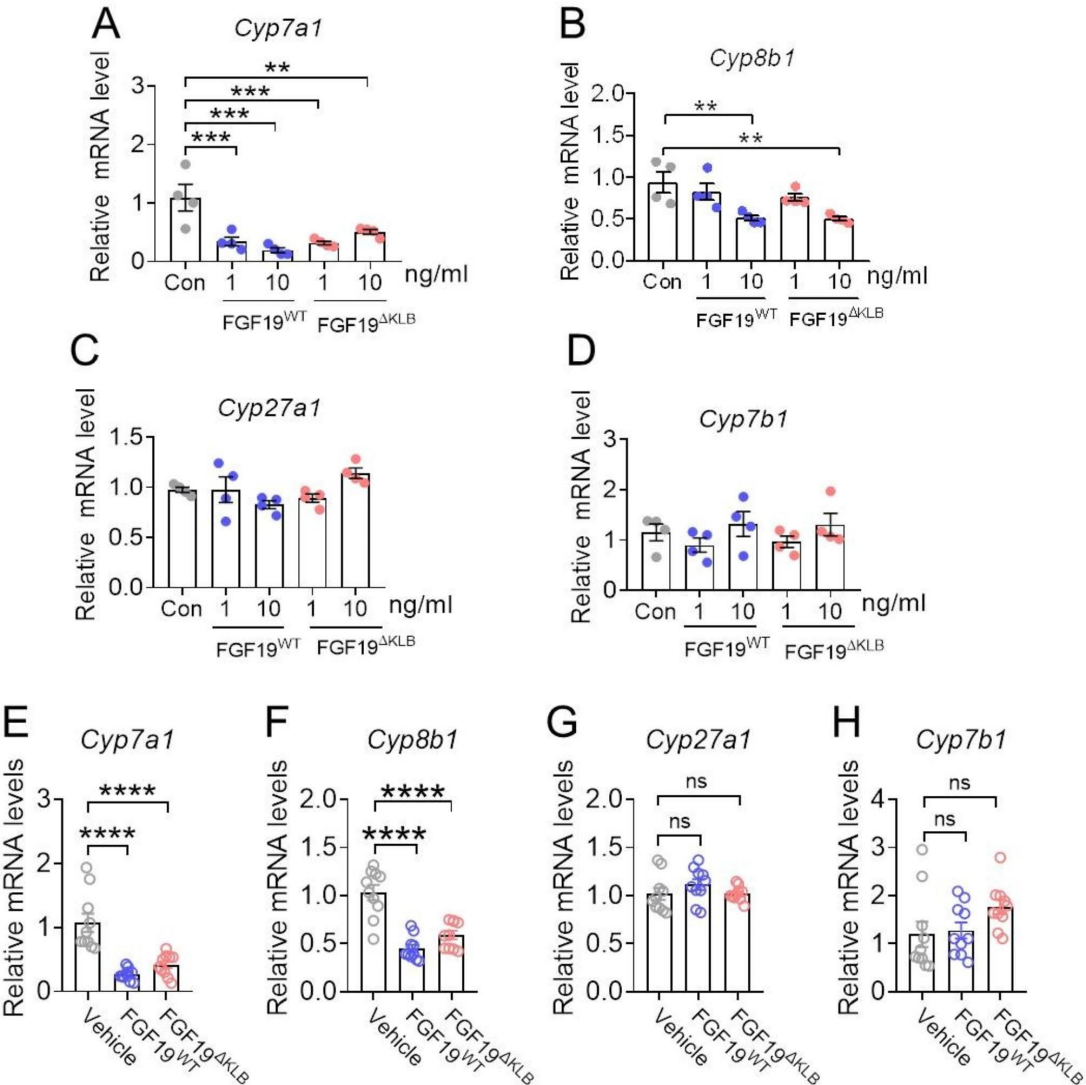
FGF19^ΔKLB^ retains BA-regulatory activity in vitro and in vivo. **(A-D)** Effects of FGF19^WT^ and FGF19^ΔKLB^ on the *Cyp7a1***(A)**, *Cyp8b1***(B)**, *Cyp27a1***(C)**, and *Cyp7b1***(D)** levels in HepG2 cell. Expression of *Cyp7a1*, *Cyp8b1*, *Cyp27a1*, and *Cyp7b1* levels were determined by RT-PCR and relative to β-actin (n = 4). **(E-H)** The effects of FGF19^WT^ and FGF19^ΔKLB^ on the hepatic *Cyp7a1***(E)**, *Cyp8b1***(F)**, *Cyp27a1***(G)** and *Cyp7b1***(H)** mRNA levels. Injections of recombinant FGF19^WT^ and FGF19^ΔKLB^ into the abdominal cavity of 12-week-old C57BL/6J mice revealed differences between the two proteins (n = 10 mice per group). RT-PCR was used to measure hepatic mRNA levels, and the results were standardized to β-actin mRNA. Mean ± SEM was chosen to represent the data; *p < 0.05, **p < 0.01, ****p < 0.0001; ns, not significant; **(A-H)** conventional one-way ANOVA, then Tukey (n = 4 or 10)

A prior work revealed that after isolating and cultivating primary mouse hepatocytes for 16 h, practically all of the *Cyp7a1* mRNA levels had been lost [28], suggesting that primary mouse hepatocytes are not suitable to study the regulation of the BA synthetic enzymes, especially Cyp7a1. The effects of various cytokines on the levels of *Cyp7a1* mRNA might be studied using HepG2 cell lines [29, 30]. Coreceptor β-Klotho and FGFR4 are endogenously expressed in HepG2 cells [31], which allows this cell line to response to FGF19 stimulation. To assess the capability of FGF19^ΔKLB^ on BA biosynthesis in *vitro*, we firstly expressed and purified recombinant FGF19^ΔKLB^ (Supplementary Fig. 1), and then investigated the gene expressions of important enzymes in BA production after stimulated by different concentrations of FGF19^ΔKLB^ in HepG2 cells. Consistent with previous studies [13, 32, 33], FGF19^WT^ stimulation drastically lowered the mRNA levels of *Cyp7a1* and *Cyp8b1*, but not *Cyp27a1* and *Cyp7a1*, in the HepG2 cells. Although FGF19^ΔKLB^ induced significantly impaired FGFR4 dimerization and downstream signaling [27], it retained comparable BA regulatory activity with FGF19^WT^ (Fig. 1A-D).

FGF19^WT^ or FGF19^ΔKLB^ (1.0 mg/kg of body weight) was injected intraperitoneally into C57BL/6J mice to further validate these in *vitro* findings in *vivo*. In line with the HepG2 findings, we discovered that *Cyp7a1* and *Cyp8b1* mRNA levels were considerably suppressed by both FGF19^WT^ and FGF19^ΔKLB^, but not by *Cyp27a1* and *Cyp7a1* (Fig. 1E-H). All of the information points to FGF19^ΔKLB^ maintaining the ability of FGF19^WT^ to control hepatic BA synthesis by inhibiting the traditional production route through *Cyp7a1* and *Cyp8b1* [34].

### FGF19^ΔKLB^ protects mice from ANIT-induced cholestatic liver injury

The etiology of cholestatic liver disease is mostly caused by hepatic buildup of BAs [35]. Delaying the course of this illness and preventing cholestatic liver damage both require reducing hepatic BA levels [36]. According to reports, FGF19 may block BA production to lower hepatic BA levels and avoid further liver damage [10].

**Fig. 2:**
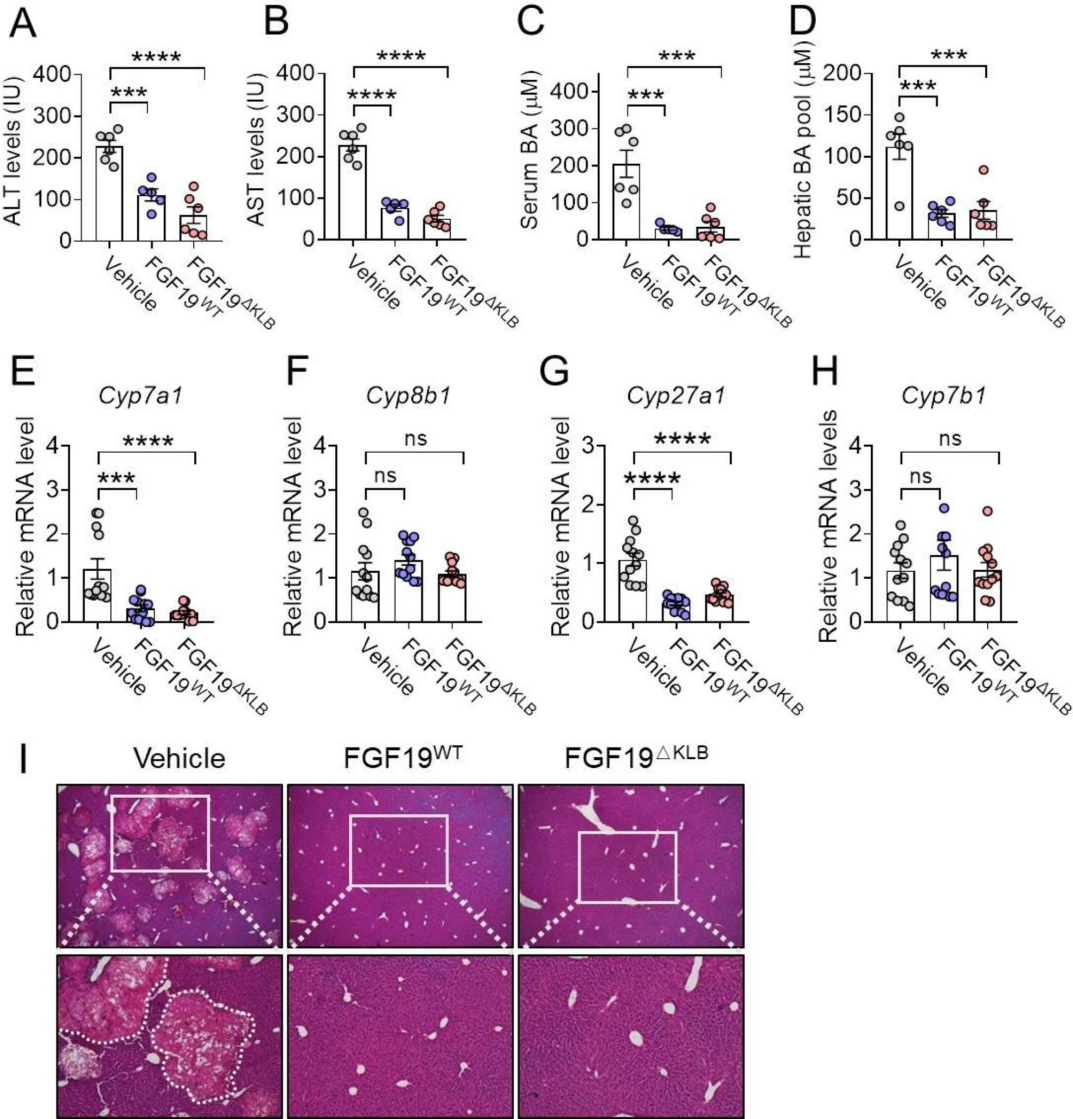
FGF19^ΔKLB^ protected against intrahepatic cholestasis brought on with ANIT. For six days, intraperitoneal injections of PBS, FGF19 (1.0 mg/kg), or FGF19^ΔKLB^ (1.0 mg/kg) were given to male C57BL/6J mice that were eight weeks old. There were six mice per group. On the fourth day, mice were orally given ANIT in olive oil (75 mg/kg) to develop an intrahepatic cholestasis animal model. As a control, six C57BL/6J male mice were not treated with ANIT. **(A-C)** Analysis of serum alanine transaminase (ALT) **(A)**, aspartate transaminase (AST) **(B)**, and total BAs **(C)** levels. **(D)** FGF19^WT^ and FGF19^ΔKLB^-treated mice exhibited a substantial reduce in the hepatic BA pool. **(E-H)** Hepatic genes expression of key enzymes in BA synthesis, including *Cyp7a1* (E), *Cyp8b1***(F)**, *Cyp27a1***(G)**, and *Cyp7b1***(H)**, was analyzed by RT-PCR. **(I)** Representative H&E-stained liver tissues. Noted that vehicle-treated ANIT mice showed multifocal liver necrosis (dashed area), while liver tissues from FGF19^ΔKLB^-treated mice had minimal or no lesions

In order to evaluate the protective effects of FGF19^ΔKLB^ in an ANIT-induced mouse intrahepatic cholestasis model, C57BL/6J mice were injected i.p. with either FGF19^WT^ or FGF19^ΔKLB^ (1.0 mg/kg of body weight) (twice daily) for a total of six days (similar to clinical primary biliary cholangitis17). The oral administration of a single dose of ANIT (75 mg/kg) occurred on day four. We found that FGF19^ΔKLB^ significantly reduced hepatic BA levels and serum total bile acid (TBA), alanine transaminase (ALT), and aspartate transaminase (AST) levels to a comparable level of FGF19^WT^, suggesting that FGF19^ΔKLB^ protected against ANIT-induced cholestatic liver injury via mitigating hepatic BA accumulation (Fig. 2A-D).

Consistent with their acute effects on C57BL/6J mice, the chronic treatments of FGF19^WT^ and FGF19^ΔKLB^ markedly inhibited hepatic *Cyp7a1* mRNA levels (the enzyme that limits the pace of the conventional BAs production pathway*)* and *Cyp27a1* (catalyzing BA biosynthesis in the alternative pathway) in ANIT-induced mouse intrahepatic cholestasis model (Fig. 2E-H). As revealed by histological analyses, swollen degeneration with multifocal necrosis and neutrophil infiltration in ANIT treated mice was largely alleviated by both FGF19^WT^ and FGF19^ΔKLB^ (Fig. 2I). Taken together, it is reasonable to speculate that FGF19^ΔKLB^ may improve intrahepatic cholestatic liver injury by reducing BA synthesis through classical and alternative pathways of BA synthesis.

### Chronic administration of FGF19 ^ΔKLB^ attenuates inflammation of the liver and liver damage in ***Mdr2***^−/−^ mice

The multi-drug resistance 2 knockout (*Mdr2*^−/−^) mouse is widely used as a model for cholestatic cholangiopathies because of the accumulation of free bile salts, which damages bile ducts and leads to fibrosis and cirrhosis in the absence of phospholipids [37]. Severe cholangiocyte damage, “onion skin”-type fibrosis, and segmental biliary strictures, dilatation were completely formed at 12 weeks in *Mdr2*^−/−^ mice with sclerosing cholangitis, which is a progressive condition. Periductal inflammation and fibrosis start at the age of four weeks [38].

**Fig. 3:**
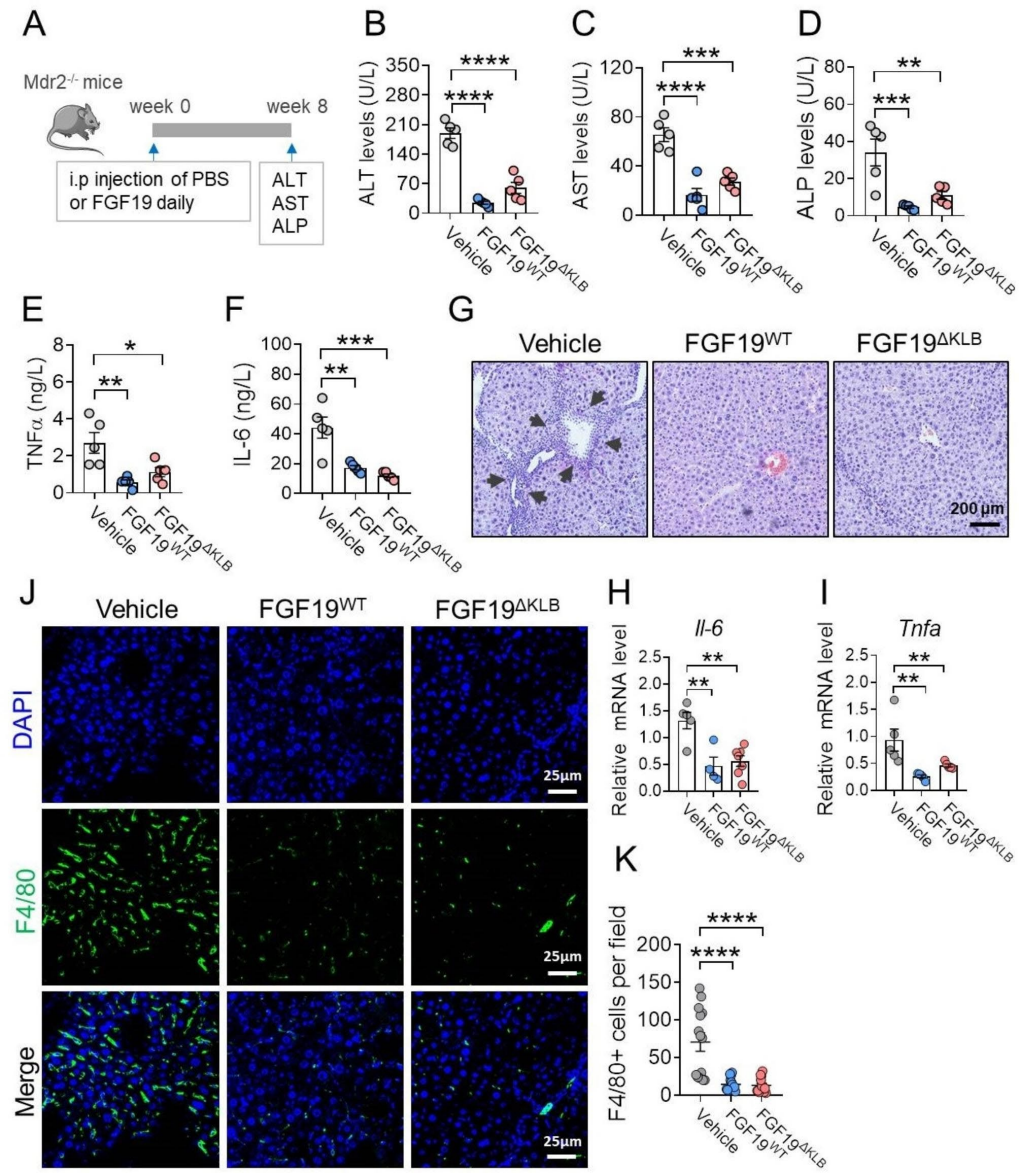
FGF19^ΔKLB^ improved hepatic functions and inflammation in Mdr2-/- mice. **(A)** Schematic diagram of experimental design. Four-week-old Mdr2-/- mice were treated by daily i.p. injections of PBS, FGF19^WT^ or FGF19 ^ΔKLB^ (n = 5 per group) for eight weeks. **(B-D)** Serum alanine transaminase (ALT) **(B)**, aspartate transaminase (AST) **(C)**, alkaline phosphatase (ALP) **(D)** levels were determined after chronic treatment. **(E-F)** Serum levels of TNFα and IL-6 were measured by ELISA. **(G)** Representative H&E staining of liver tissues in Mdr2^−/−^ mice treated by PBS, FGF19^WT^ or FGF19^ΔKLB^. **(H-I)** Hepatic pro-inflammatory cytokines including Il6 **(H)** and TNF-α **(I)** mRNA levels. **(J-K)** Immunofluorescence **(J)** and semi-quantification **(K)** of F4/80 positive cells in liver tissues. Mean ± SEM was chosen to represent the data; **p < 0.01, ***p < 0.001, ****p < 0.0001; **(B-D, F, G, I)** conventional one-way ANOVA, then Tukey

To evaluate the therapeutic effects of FGF19^ΔKLB^ on *Mdr2*^−/−^ mice, four-week-old mice received daily intraperitoneal injections of FGF19^WT^ or FGF19^ΔKLB^ (1.0 mg/kg body weight) for eight weeks (Fig. 3A). We discovered that the serum ALT, AST, and ALP levels were markedly reduced in both FGF19^WT^ and FGF19^ΔKLB^-treated mice (Fig. 3B-D). In addition, serum levels of pro-inflammatory markers tumor necrosis factor alpha (TNFα) and interleukin-6 (IL-6) levels were also significantly decreased (Fig. 3E&F). These data indicated that liver injury in *Mdr2*^−/−^ was alleviated by the chronic administration of this engineered FGF19 mutant.

Bile in *Mdr2*^−/−^ mice flows back from leaky duct to portal tract area, leading to periductal inflammation [37], this is essential for the development of sclerosing cholangitis. We next analyzed the effects of FGF19^ΔKLB^ on hepatic inflammation in *Mdr2*^−/−^ mice. The histological analyses showed that chronic FGF19^ΔKLB^ and FGF19^WT^ treatment significantly mitigated infiltration of neutrophils around central vein and alleviated bile duct hyperplasia in the liver compared to vehicle treatment (Fig. 3G). Consistently, the immunofluorescence analyses showed a reduced F4/80 positive macrophage infiltration by FGF19^ΔKLB^ and FGF19^WT^ treatment with reduced the pro-inflammatory markers tumor necrosis factor alpha (TNFα) and interleukin-6 (IL-6) levels (Fig. 3H-K). All of these findings point to the fact that prolonged FGF19^ΔKLB^ treatment in *Mdr2*^−/−^ mice reduces liver inflammation and damage.

### Chronic FGF19^ΔKLB^ treatment alleviates hepatic fibrosis in ***Mdr2***^−/−^ mice

**Fig. 4:**
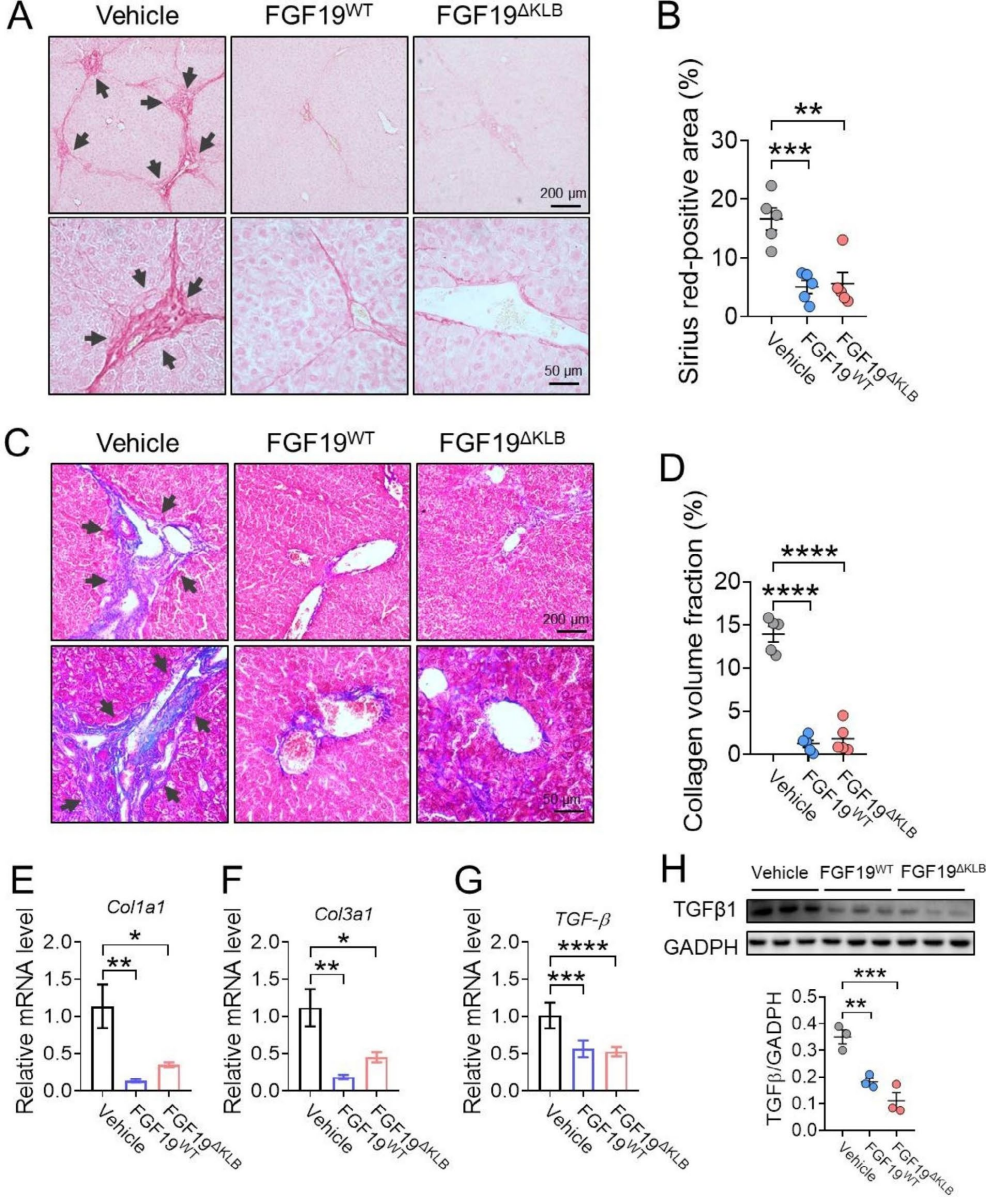
FGF19^ΔKLB^ exerted anti-fibrotic effect in the liver tissues of Mdr2-/- mice. Four-week-old Mdr2^−/−^ mice were treated by daily i.p. injections of PBS, FGF19^WT^ or FGF19 ^ΔKLB^ (n = 5 per group). **(A-B)** Representative images of Sirius red staining **(A)** of livers from Mdr2-/- mice treated with PBS, FGF19^WT^ or FGF19 ^ΔKLB^ for eight weeks and its semi-quantification **(B)**. **(C-D)** Masson’s trichrome staining **(C)** to evaluate collagen levels (black arrows) and collagen volume fractions are determined semi-quantatively by Image J **(D)**. When stained with Sirius Red or Trichrome, the collagen fibers in the periduct look red. **(E-F)** Hepatic mRNA levels of the pro-fibrotic genes Col1a1 **(E)** and Col1a2 **(F)** were evaluated by RT-PCR. **(G-H)** mRNA **(G)** and protein **(H)** levels of TGF-β **(G)**. Mean ± SEM was chosen to represent the data; *p < 0.05, **p < 0.01, ***p < 0.001, ****p < 0.0001; **(C, D, E-H)** conventional one-way ANOVA, then Tukey. The original blot image is shown in Figure S1

Underlying progression of inflammation and proliferation in bile ducts may result in biliary fibrosis [39], we therefore assessed the effects of chronic FGF19^ΔKLB^ and FGF19^WT^ treatment on liver fibrosis in *Mdr2*^−/−^ mice. As shown by investigations of Masson staining and Sirius red, periductal collagen deposition (as indicated by the arrow) was largely compromised by FGF19^ΔKLB^ and FGF19^WT^ compared to vehicle treatment (Fig. 4A-D). *Col1a1* and *Col3a1* were two fibrosis indicators with dramatically reduced mRNA levels by FGF19^ΔKLB^ and FGF19^WT^ treatment (Fig. 4E-F). In addition, the transcription and protein levels of TGF-β (a pro-fibrotic cytokine [40]) were reduced by FGF19^ΔKLB^ and FGF19^WT^ treatment (Fig. 4G-H). All of these findings together show that chronic administration of FGF19^ΔKLB^ exerts an anti-fibrotic effect on the liver of *Mdr2*^−/−^ mice as FGF19^WT^.

### FGF19^ΔKLB^ restores BA homeostasis in ***Mdr2***^−/−^ mice

**Fig. 5:**
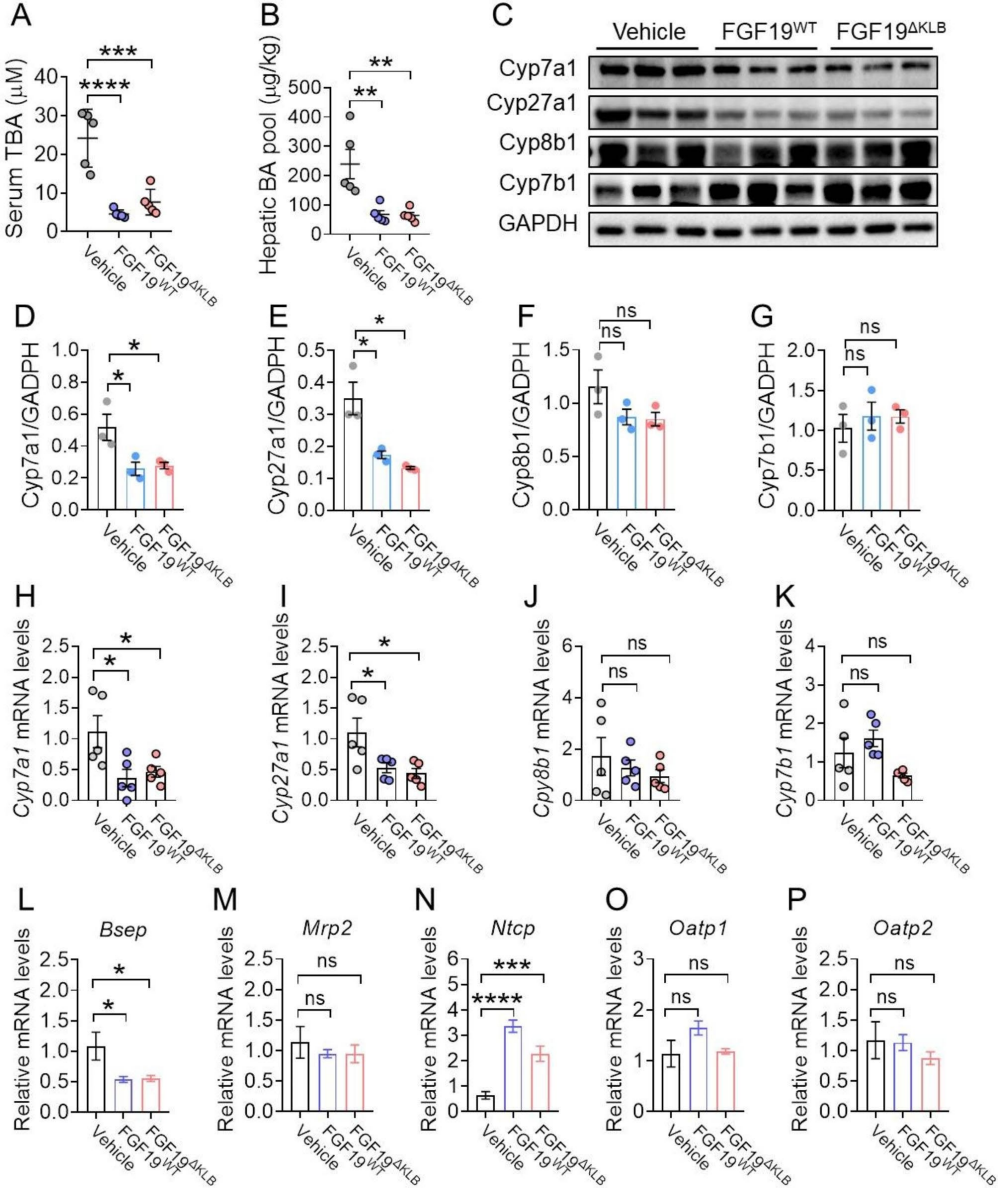
FGF19^ΔKLB^ inhibited BA biosynthesis and regulated BA homeostasis. Four-week-old Mdr2^−/−^ mice were treated by daily i.p. injections of PBS, FGF19^WT^ or FGF19^ΔKLB^ (n = 5 per group) for eight weeks. **(A-B)** Serum total BA (TBA) **(E)** and hepatic BA pool **(F)** levels after treated by FGF19^WT^ or FGF19. **(C-G)** Western blotting analysis of hepatic Cyp7a1, Cyp27a1, Cyp8b1 and Cyp7b1 protein expression levels (C) and its semi-quantification **(D-G)**. **(H-K)** Hepatic *Cyp7a1* (H), *Cyp27a1***(I)**, *Cyp8b1***(J)**, and *Cyp7b1***(K)** mRNA levels were evaluated by RT-PCR. **(L-P)** Expression profiles of BA canalicular efflux transporter (*Bsep* and *Mrp2*) **(L-M)** and basolateral uptake transporter (Ntcp, Oatp1 and Oatp2) **(N-P)** were determined by RT-PCR. Mean ± SEM was chosen to represent the data; *p < 0.05, **p < 0.01, ***p < 0.001, ****p < 0.0001; ns, not significant; **(A-D, F-M)** conventional one-way ANOVA, then Tukey

Serious cholestasis is mostly caused by dysregulation of BA metabolism brought on by Mdr2 impairment, which results in high levels of BAs in the blood [38]. We observed that serum level of TBA and hepatic BA levels were significantly reduced by treatment of FGF19^WT^ and FGF19^ΔKLB^ compared to that of vehicle-treated group (Fig. 5A-B). Then, we looked at how they affected the expression of vital BA synthesis enzyme genes. Similar with the results observed in mouse model for intrahepatic cholestasis brought on by ANIT (Fig. 2E-H), RT-PCR and Western blotting analysis showed that both FGF19^ΔKLB^ and FGF19^WT^ significantly inhibited the mRNA and protein levels of hepatic Cyp7a1 (the rate-limiting enzyme in the classical pathway) and Cyp27a1 (catalyzing BA biosynthesis in the alternative pathway), without alter Cyp8b1 and Cyp7b1 (Fig. 5C-K). Therefore, all these data suggest that FGF19^ΔKLB^ administration reduces BA synthesis and improves hepatic BA pool to a similar level as that of FGF19^WT^.

Couples of hepatic BA transporters are essential for maintaining the homeostasis of BA in addition to regulating its production [38]. Basolateral uptake transporters contribute much to the liver’s ability to absorb BA from the hepatic portal vein (including Oatp2, Oatp1, and Ntcp), although BA canalicular efflux transporters, who control its excretion primarily, including Bsep, Mrp2 and Mdr2 [41]. We therefore analyzed the effects of FGF19^WT^ and FGF19^ΔKLB^ treatment on mRNA levels of BA transporters, and found that transcription levels of hepatocellular uptake transporters and canalicular efflux pumps were not affected by FGF19^WT^ and FGF19^ΔKLB^ (Fig. 5L-P). Taken together, all the data suggest that chronic FGF19^ΔKLB^ treatment reduces hepatic BA accumulation and maintains its homeostasis via suppressing BA synthesis in the *Mdr2*^−/−^ mice’s liver.

### FGF19^ΔKLB^ did not induce proliferation in the ***Mdr2***^−/−^ mice’s liver

**Fig. 6:**
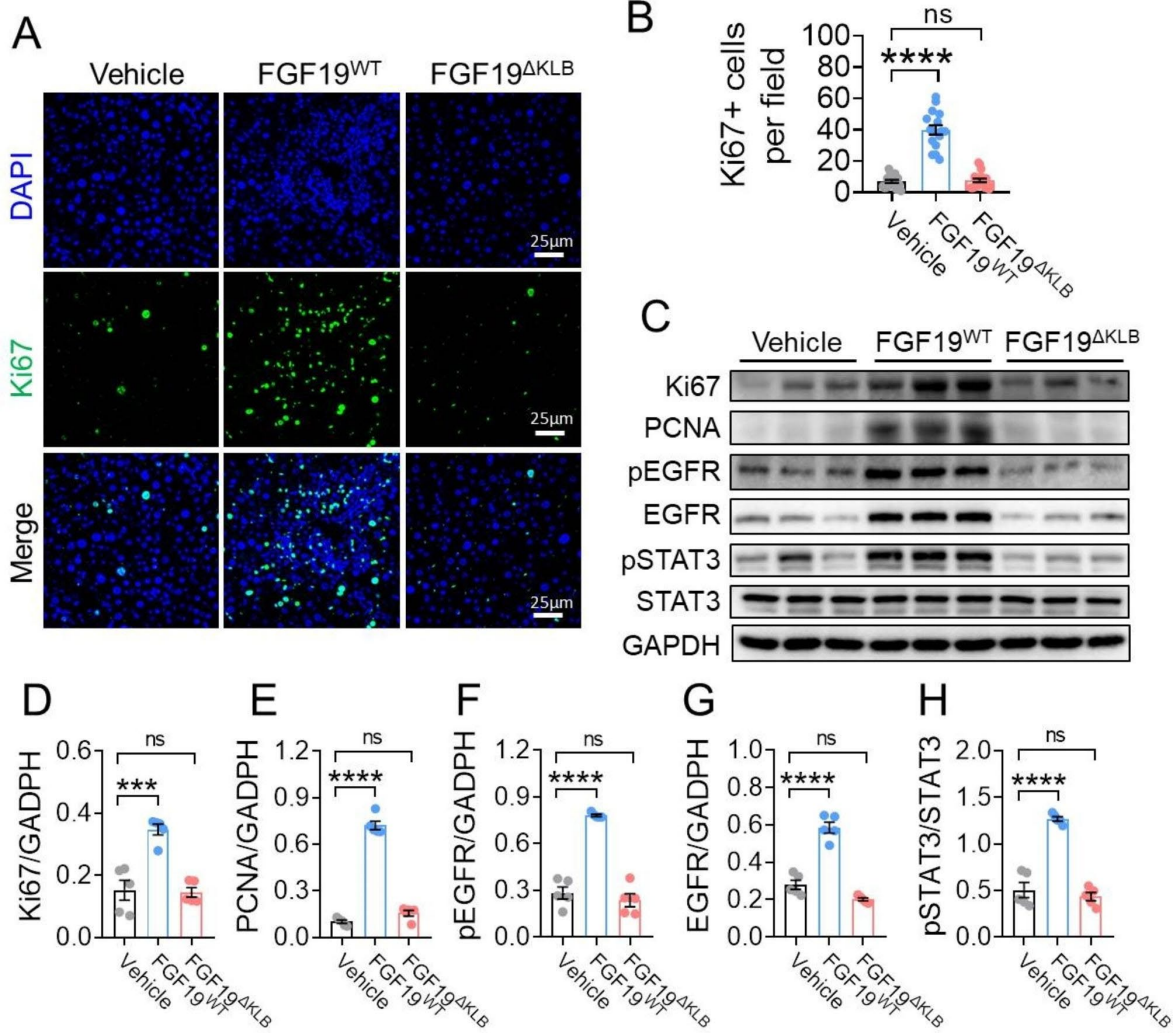
FGF19^ΔKLB^ did not induce hepatic proliferation and cancer-associated signaling pathways. Four-week-old Mdr2^−/−^ mice were treated by daily i.p. injections of PBS, FGF19^WT^ or FGF19^ΔKLB^ (n = 5 per group) for eight weeks. **(A-B)** Representative images of Ki67 immunofluorescence staining **(A)** of livers from Mdr2-/- mice treated with PBS, FGF19^WT^ or FGF19 ^ΔKLB^ for eight weeks and its semi-quantitation. Image J was used to check for Ki67-positive hepatocytes on two to three pictures of randomly chosen regions from five different mice per group **(B)**. **(C-H)** Hepatic protein expression of Ki67, PCNA, pEGFR, EGFR, pSTAT3 and STAT3 **(C)** as evaluated by Western blotting and its semi-quantitation **(D-H)** using Image J. (**B, D-H**) Mean ± SEM was chosen to represent the data; ***p < 0.001, ****p < 0.0001; conventional one-way ANOVA, then Tukey. The original blot image is shown in Figure S2

To evaluate the safety of chronic FGF19^ΔKLB^ treatment on *Mdr2*^−/−^ mice, the protein expressions of proliferation marker including Ki67 and proliferating cell nuclear antigen (PCNA) were analyzed. We found that upregulated protein levels of Ki67 and PCNA by FGF19^WT^ were largely compromised in the liver of mice treated with FGF19^ΔKLB^ (Fig. 6A-E). In addition, we also looked at the levels of p-EGFR, EGFR, p-STAT3, and total STAT3 protein expression, which are important signaling pathways involved in liver fibrosis and hepatocellular carcinoma (Fig. 6C-H). In contrast with FGF19^WT^, FGF19^ΔKLB^ did not activate EGFR signaling pathways (Fig. 6C, F, G). We also observed that consistent with previous reports, FGF19^WT^ treatment induced STAT3 phosphorylation rather than increased STAT3 protein level FGF19^WT^ (Fig. 6C, H), while structural optimization makes FGF19^ΔKLB^ evade the activation of STAT3 signaling pathway. In summary, during the investigation period, long-term injection of FGF19 did not induce liver safety risk and probably a potential substitute for FGF19^WT^ in practical application.

Previous reports showed that *db/db* mice exhibited the shortest latency and higher tumor incidence among several tested mouse strains after AAV-mediated overexpression of FGF19, which provides a robust system to evaluate FGF19-induced hepatocarcinogenesis in vivo [20, 42]. Based on this, we overexpressed FGF19^WT^ and FGF19^ΔKLB^ for 24 weeks in *db/db* mice using the AAV-mediated delivery system. At the end of this experiment, visible tumor nodules were counted on the entire liver surface and hepatic histological analysis was performed. The results showed that, compared with that of FGF19^WT^, FGF19^ΔKLB^ failed to induce the hepatocellular carcinoma, as revealed by macroscopic morphology, liver index ( the ratio of liver weight to body weight), the number of tumors per liver and histological examination (Supplementary Fig. 2), further confirming the safety of FGF19^ΔKLB^ under the chronic treatment.

## Discussion

Ursodeoxycholic acid (UDCA), as a first-line treatment drug for cholestatic liver injury, can indeed improve the hepatic function of primary sclerosing cholangitis (PSC) patients, but 30–50% of patients failed to respond this treatment [43]. Therefore, alternative pharmacological therapeutics is urgently warranted for those with insufficient benefit from UDCA. In this study, we demonstrate that FGF19^ΔKLB^, an FGF19 analogue engineered in our recent study, exerts potent hepatoprotective activity in two intrahepatic cholestatic mouse models, ANIT-induced and *Mdr2*^−/−^ mice. FGF19^ΔKLB^ retains the ability of FGF19 to regulate BAs homeostasis via suppressing its synthesis. Interestingly, FGF19^ΔKLB^ also shows strong anti-inflammatory and anti-fibrosis properties on the liver of *Mdr2*^−/−^ mice, indicating that this analogue protects bile duct epithelial cells from damage via multiple ways besides its regulation of BAs homeostasis. Taken together, this study indicates that FGF19^ΔKLB^ probably be an effective medication that might be used to treat cholestatic liver damage.

As a key downstream target of the nuclear transcription receptor FXR, endocrine FGF19 showed potent regulatory activity of BAs and postprandial lipid and glucose metabolism, which sparks interest in clinical translation of FGF19 or its analogues for cholestatic liver disease and nonalcoholic steatohepatitis (NASH) [44]. However, hepatocellular carcinoma induced by chronic administration of FGF19^WT^ becomes the major safety concern that hinders its clinical development [15–17]. By lowering FGF19’s ability to dimerize its corresponding FGFRs, we were able to generate non-mitogenic FGF19 molecules, supporting our “threshold model” hypothesis that FGF signaling specificity is regulated by distinct thresholds in FGF-induced FGFR dimer stability and longevity [27, 45]. In particular, the Asp-198-Ala mutation in FGF19^ΔKLB^ interferes with the binding of its coreceptor, klotho (KLB), by destroying intramolecular hydrogen bonds. The resulting FGF19^ΔKLB^ maintained all of FGF19’s favorable glucose-lowering and BA regulating functions. The FGFR4-meidated BA metabolic signaling pathway was not activated by the previously described non-tumorigenic FGF19 variants, which were created specially to prevent binding and activation of FGFR4 ^27^. M70/NGM282, a FGF19 analogue with three amino acid alterations (A30S, G31S, and H33L) and a five amino acid deletion in the N-terminal, efficiently suppresses hepatic BA synthesis without elucidating the molecular underpinnings of these variants’ non-mitogenic properties [38]. In contrast to the FGF19^ΔKLB^ used in this study, M70 did not exert any glycemic control because it did not adequately stimulate the adipose-tissue localized FGFR1c pathway [27, 38].

Although our data of preclinical mouse experiments showed that the engineered FGF19^ΔKLB^ exerted a potent therapeutic effect on cholestatic liver injury and was expected to become a promising candidate for this disease, this research has limitations that need to be addressed. First, there were only two mice models of intrahepatic cholestatic liver damage employed as preclinical models in this investigation. Clinically, cholestatic liver injury mainly includes intrahepatic cholestasis induced by secretion disorder of hepatocytes or cholangiocytes and extrahepatic cholestatic liver injury caused by blockage of the bile ducts mechanically, including gallstones, bile duct or pancreatic carcinoma and choledochal stricture end [36]. Therapeutic effects of FGF19^ΔKLB^ on the cholestasis has not been verified in more widely established experimental mode, such as surgically created 3,5-diethoxycarbonyl-1,4-dihydrocollidine (DDC) and bile duct ligation (BDL) mice or lithocholic acid-fed mice. Second, a more systematic and in-depth toxicology experiment upon chronic administration in the rodent and health human should be performed to confirm the safety of this engineered FGF19^ΔKLB^.

## Conclusions

In conclusion, we found that FGF19^ΔKLB^, an engineered FGF19 analogue, ameliorated the development of liver damage and fibrosis in intrahepatic cholestatic mouse models by improving abnormal accumulation of BAs and inflammation. These findings suggested that FGF19^ΔKLB^ may be a potential therapeutic candidate for cholestatic liver disease.

## Methods

### Cell based experiments

HepG2 cells (5.5 × 10^5^ cells/cm^2^) from the Shanghai Cell Bank were identified by short tandem repeat (STR) (Supplementary Fig. 3) and planted on 6-well plates for a period of 24 h, where they were then allowed to adhere and grow for a further 24 h in an incubator (37 °C, 5% CO_2_). Williams’ E medium with fetal bovine serum (2%), L-glutamine, 1% penicillin/streptomycin, and 10 nM dexamethasone made up the culture media. Cells were exposed to FGF19 and FGF19^ΔKLB^ (1.0 or 10 nM) for 6 h after being isolated for 12 h. *Cyp7a1*, *Cyp8b1*, *Cyp27a1* and *Cyp7b1* mRNA levels were extracted using Trizol reagent (Invitrogen) and quantified by real-time polymerase chain reaction (RT-PCR).

### Expression and purification of recombinant FGF19^WT^ and FGF19^ΔKLB^

Briefly, a DNA fragment encoding FGF19^WT^ or FGF19^ΔKLB^ was subcloned into the bacterial expression vector pET28a. Constructs were transformed into *Escherichia coli BL21* (DE3). Protein expression was induced with 1 mM isopropyl β-D-1-thiogalactopyranoside (IPTG) at 37 °C for 4 h and the cells were collected by centrifugation. FGF19^WT^ or FGF19^ΔKLB^ was refolded in vitro from isolated bacterial inclusion bodies using published protocols [46, 47]. Then, refolded FGF19^WT^ or FGF19^ΔKLB^ containing an N-terminal histidine tag was purified by nickel affinity column (HisTrap HP) and size exclusion chromatography (HiLoad 16/600 Superdex 75 column) with an AKTA purifier (GE Healthcare). The purity of FGF19^WT^ or FGF19^ΔKLB^ was estimated to be greater than 95% based on SDS-PAGE analysis. Protein concentrations were determined by Nanodrop.

### Animals and animal welfare

Zhejiang Vital River Experimental Animal Technology Co. LTD. sold us male C57BL/6J mice weighing 20–25 g. Jackson Laboratory offered *Mdr2*^−/−^ mice on an FVB/N background (stock number 002539) in addition to sex- and age-matched wild-type FVB/N mice. These Mice were raised at Wenzhou Medical University, China, and housed in a specific pathogen-free animal facility in a controlled environment and given free access to food and water. All animal care practices and research were approved by the Wenzhou Medical University in China’s Animals Care and Use Committee. At the end of the experiments, the mice were anesthetized with intraperitoneal injection of 150–200 mg/kg amobarbital sodium and sacrificed by cervical dislocation. Tissues samples were harvested for subsequent analyses.

### Acute effects of FGF19 on the enzymes essential for BA biosynthesis

To assess the acute effects of FGF19^WT^ and FGF19^ΔKLB^ on the major enzymes in BA biosynthesis, C57BL/6J mice were given an intraperitoneal injection of PBS, FGF19^WT^ and FGF19^ΔKLB^ (1.0 mg/kg, n = 10 animals for each treatment). After four hours of dosing, liver tissue was obtained. Using RT-PCR, hepatic mRNA levels were determined and adjusted to β-actin mRNA levels.

### Blood parameters measurement

Blood was collected after mouse death using EP tubes. Alkaline phosphatase (ALP) (A059-2), aspartate transaminase (AST) (C010-2-1), and alanine transaminase (ALT) (C009-2-1) were obtained from Nanjing Jiancheng Bioengineering Institute and measured on a microplate reader (MX190). According to the instructions provided by the manufacturer, all tests were completed.

### FGF19’s therapeutic impact on intrahepatic cholestasis brought on by ANIT

Based on body weight (n = 6), three groups of mice were created and intraperitoneally injected once daily for six days with PBS, FGF19^WT^ (1.0 mg/kg), and FGF19^ΔKLB^ (1.0 mg/kg). A single oral dose of ANIT (75 mg/kg body weight, dissolved in olive oil) was given to mice on the fourth day. On the sixth day, 4 h after the last dose, the mice were put to death. Blood samples were collected via the retro-orbital route for subsequent liver function test. Liver tissues were collected for subsequent experiments.

### Therapeutic effects of FGF19 in the ***Mdr2***^−/−^ mice

*Mdr2*^−/−^ mice that were four weeks old were given daily intraperitoneal injections of PBS, FGF19^WT^ or FGF19 ^ΔKLB^ (1.0 mg/kg, n = 5 per group) for eight weeks. Blood samples were collected via the retro-orbital route for subsequent liver function test. Liver tissues were collected for subsequent experiments.

### Hepatic bile acid pool size and serum BA concentration measurements

Total BAs in the liver were quantified using the Mouse Total BA Assay Kit (Crystal Chem INC). Total BAs were extracted from the homogenate by agitating the tissue for two hours at 50 °C after it had been homogenized with 75% ethanol from individual livers. After being centrifuged, the extraction’s supernatants were collected and diluted in PBS for examination. The dilution parameters for each tissue extract were determined to ensure that the BA readings obtained using a mouse BA kit were within the linear range of the standard curve. The liver’s total number of BAs was added to estimate the size of the BA pool.

### An AAV-mediated delivery system to evaluate the tumorigenesis of FGF19

AAV-FGF19^WT^ and AAV-FGF19^ΔKLB^ vectors purchased from GeneChem Co., Ltd (Shanghai, China) were injected into eight-week-old *db/db* mice (2*10^11^ vector genomes per mice) via tail vein for 24 weeks. At the end of the experiment, liver tissue was collected, and analyzed for macroscopic morphology and liver index (the ratio of liver weight to body weight). The number of tumors per liver from *db/db* mice was counted. H&E staining and immunohistochemical staining using glutamine synthetase (GS), Ki67 and PCNA of liver tumors in *db/db* mice were performed.

### Histological analysis

Formalin-fixed and implanted mouse liver was used. Sections of paraffin (5 μm) were produced for Masson’s Trichrome (G1346, Solarbio), hematoxylin and eosin (HE) staining (G1120, Solarbio), and Picro-Sirius Red (DC0041, Leagene Biotechnology) staining were used to evaluate liver fibrosis. Using a light microscope, histological pictures of tissue slices were taken (Nikon eclipse Ni, Tokyo, Japan).

### Immunofluorescent staining

Liver slices were deparaffinized with xylene after being roasted for 5 h at 65 °C, and then rehydrated in a series of ethanol solutions of gradually decreasing concentrations. Livers were sliced, washed in PBS, and then boiled in a 10 mM sodium citrate buffer for 2 min at 100 °C (pH 6.0). Liver slices were blocked for 1 h with 5% BSA after being treated with 3% H_2_O_2_ for 30 min and then washed twice in PBS. After that, liver slices were incubated with primary antibodies for F4/80 (ab60343, Abcam) and Ki67 (ab16667, Abcam) for an entire night at 4 °C. After a PBS wash, slices were given a second, three-time PBS wash before being incubated with the Alexa Fluor 488-conjugated secondary antibody (ab15007, Abcam) for one hour. Nuclei were stained in contrast using DAPI. Using a Nikon C2si Confocal Microscope, fluorescent pictures were acquired. Using ImageJ, data were quantified.

### Real-time polymerase chain reaction (RT-PCR)

From frozen liver tissues, total RNA was extracted with a TransZol Up Kit (ET111-01, TransGen Biotech) and was quantified using a NanoDrop One spectro- photometer (Thermo Fisher). Then, a One-Step gDNA Removal Kit was chosen to reverse-transcribe 2 µg of RNA into cDNA (AT341, TransGen Biotech). While performing at a Level One Plus The specific primers listed in Supplementary Table 1 were used for quantitative RT-PCR utilizing Real-Time PCR equipment (Applied Biosystems® Quant Studio® 3) and the ChamQ Universal SYBR qPCR Master Mix (Q711, Vazyme).

### Western blot analysis

After centrifugation, the homogenate containing the liver tissues was washed with phosphatase and protease inhibitors in protein extraction buffer (Sigma-Aldrich, St. Louis, MO). To determine total protein concentration, a bicinchoninic acid (BCA) protein assay kit (Thermo Scientific, Waltham, MA) was used. Electrotransfer of proteins from SDS-polyacrylamide gels to PVDF membranes (Millipore-Billerica, MA, USA). Then, the PVDF membranes were cut based on the molecular weight of the target proteins, prior to hybridisation with antibodies. Tris-buffered saline (pH 7.6) containing 0.1% Tween-20 (TBST) was used to wash the PVDF membranes three times after they were blocked in 10% nonfat milk for an hour at room temperature and then blots were incubated with primary antibodies against glyceraldehyde-3-phosphate dehydrogenase (GAPDH) (CST2118, Cell Signaling Technology); Cyp7a1 (ab65596, Abcam); Cyp8b1 (ab191910, Abcam), Cyp27a1 (ab126785, Abcam), Cyp7b1 (ab138491, Abcam), TGFβ1 (ab824, Abcam); Ki67 (ab16667, Abcam); PCNA (ab29, Abcam); phosphorylated EGFR (CST2231, Cell Signaling Technology); EGFR (CST4267, Cell Signaling Technology); phosphorylated STAT3 (CST9145, Cell Signaling Technology); STAT3 (CST8768, Cell Signaling Technology) at 4 °C overnight. After being washed, membranes were incubated with a secondary antibody labeled with horseradish peroxidase (HRP) for 1 h at room temperature. An enzyme called glyceraldehyde 3-phosphate dehydrogenase (GAPDH) was employed to make sure everything was running well. The ChemiDocTM XRS coupled with Imaging LabTM Software was used for the visualization of immunoreactive protein bands (Bio-Rad, Hercules, CA). The protein concentrations were normalized with respect to GAPDH using Image J (National Institutes of Health, Bethesda, MD, USA).

### Analytical statistics

The statistical analysis was performed using GraphPad Prism 8. In studies with just two groups, the means were compared using either the Mann-Whitney U test or a two-tailed Student’s *t*-test. When comparing data from different groups, we utilized one- or two-way ANOVA (ordinary or repeated measure) with post-hoc test (Tukey or Sidak) as appropriate. A p-value of less than 0.05 was considered significant.

### Electronic supplementary material

Below is the link to the electronic supplementary material.

Supplementary Material 1

## Data Availability

All data generated or analyzed during this study are included in this published article and its supplementary information files.
