# Supplementary material for: Engineered FGF19ΔKLB protects against intrahepatic cholestatic liver injury in ANIT-induced and Mdr2-/- mice model
Source: BMC Biotechnol. 2023 Oct 3;23:43. doi: 10.1186/s12896-023-00810-9 (PMC10548598; doi:10.1186/s12896-023-00810-9)
Supplement: Supplementary file 1 — Supplementary Material 1 [file 12896_2023_810_MOESM1_ESM.docx]

Supplementary Materials

**
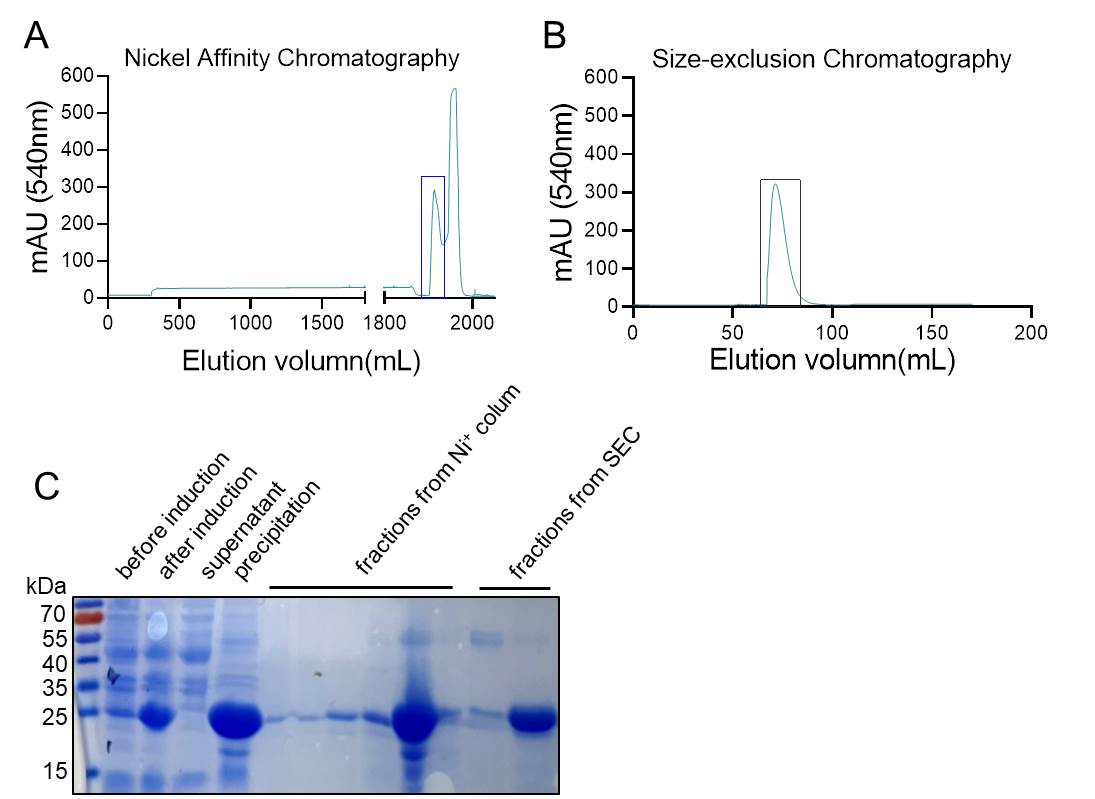
**

**Figure S1.** Refolded FGF19^ΔKLB^ containing an N-terminal His tag was purified by nickel affinity column (A) and size exclusion chromatography (B). (C) SDS-PAGE was used to analyze the samples involved in the expression and purification of FGF19^ΔKLB^, including *Escherichia coli* before and after IPTG induction, the supernatant and precipitation of *Escherichia coli* after lysis, the fractions from nickel affinity chromatography and size exclusion chromatography (SEC). In order to minimize the content of endotoxin in the protein samples, all the water used in this study is double distilled water and all relative containers were strictly sterilized.

**
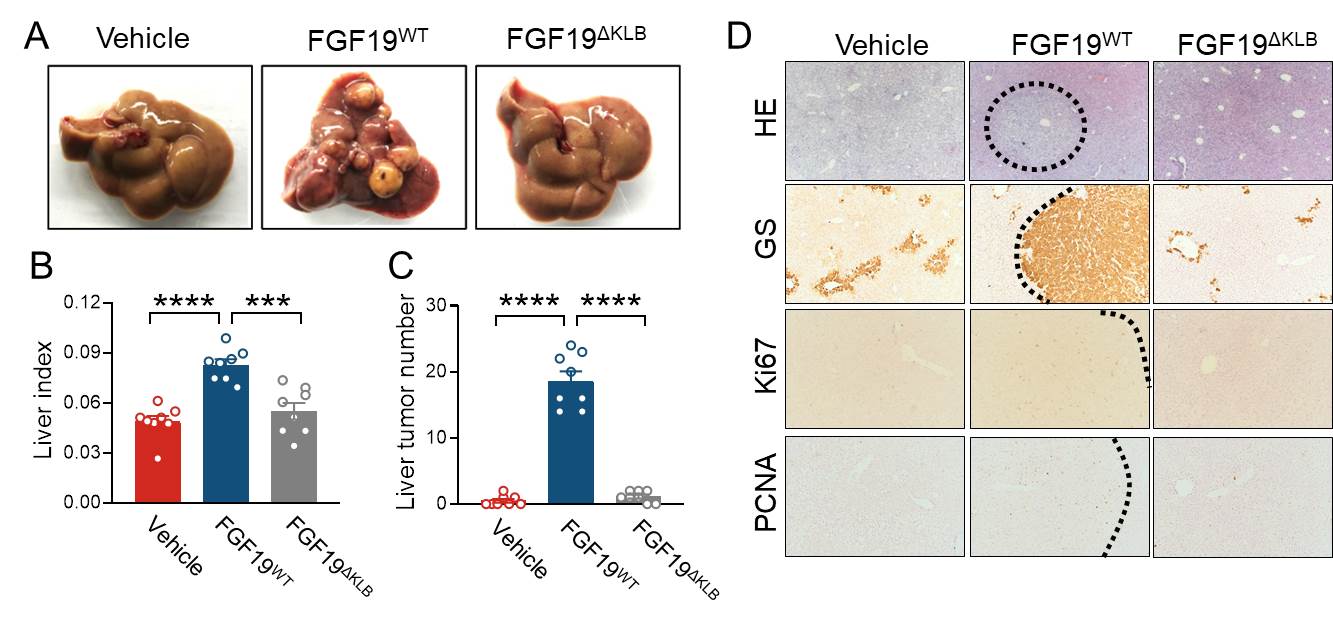
**

**Figure S2** An AAV-mediated delivery system to evaluate the tumorigenesis of FGF19^WT^ and FGF19^ΔKLB^. AAV-FGF19^WT^ and AAV-FGF19^ΔKLB^ vectors (2*10^11^ vector genomes per mice) were injected into eight-week-old *db/db* mice via tail vein for 24 weeks (n=8). At the end of the experiment, liver tissue was collected and analyzed for macroscopic morphology (A), liver index ( the ratio of liver weight to body weight, B), and the number of tumors per liver (C). (D) H&E staining and immunohistochemical staining using glutamine synthetase (GS), Ki67 and PCNA of liver tumors from *db/db* mice. Tumors are outlined by dotted lines. Data are presented as mean values ± SEM; ***p<0.001; ****p < 0.0001.


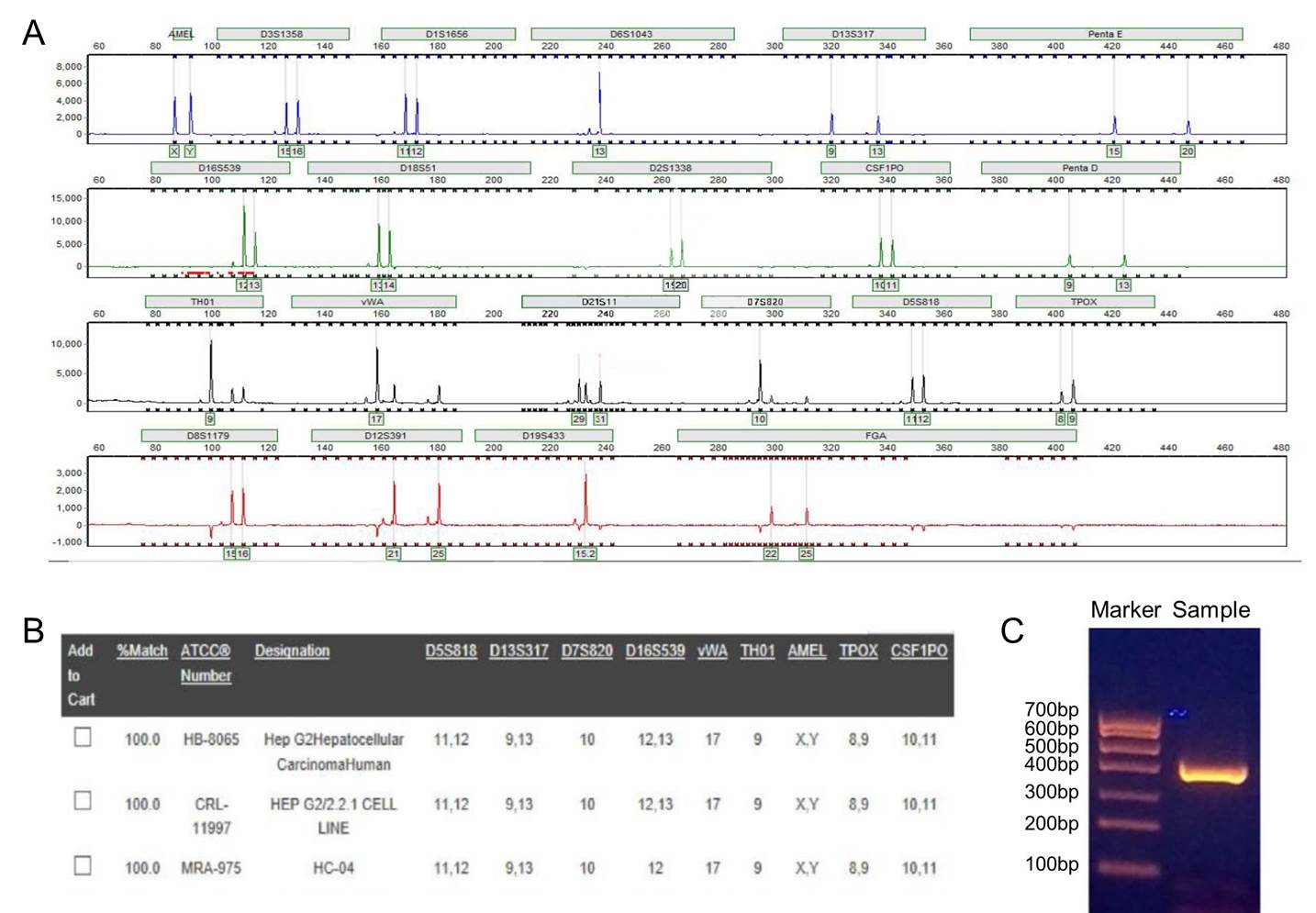


**Figure S3** HepG2 cells were identified by short tandem repeat (STR). The STR profiles of HepG2 cells used in our experiments obtained by using STR Multi-amplification Kit (PowerPlex 21 System). (A) The STR profiles of the cell lines showed that no loci has tri-alleles or tetra-alleles, and contamination of other human cell lines is not found. (B) Search result in the databases of American Type Culture Collection (ATCC) showed that all the alleles of HepG2 cells used in our experiments were exactly matched with the alleles of HepG2 cells in the ATCC. (C) Electrophoretic analysis of the species of the samples. Lane Marker: DNA ladder; lane Samples: HepG2 cells used in our experiments. The band size of HepG2 cells used in our experiments is about 391 bp which matched the size of human.

**
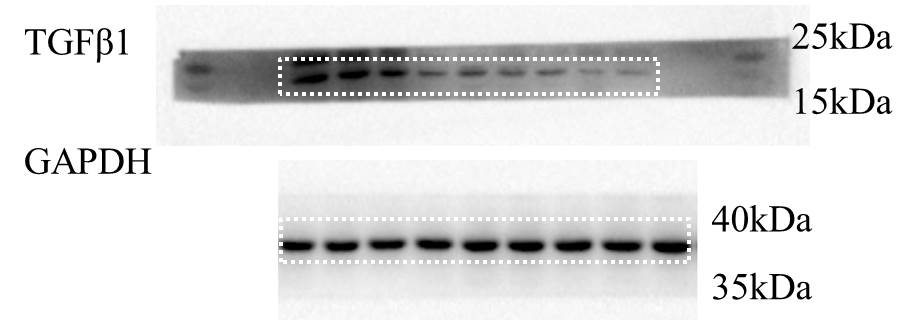
**

Figure S4.The original blot image of Figure 4H. Western blot analysis of livers from Mdr2-/- mice treated with PBS, FGF19^WT^ or FGF19^ΔKLB^ using anti-TGFβ antibody. All cropped blot image parts in the manuscript are highlighted with white dashed frames on the original blot images.


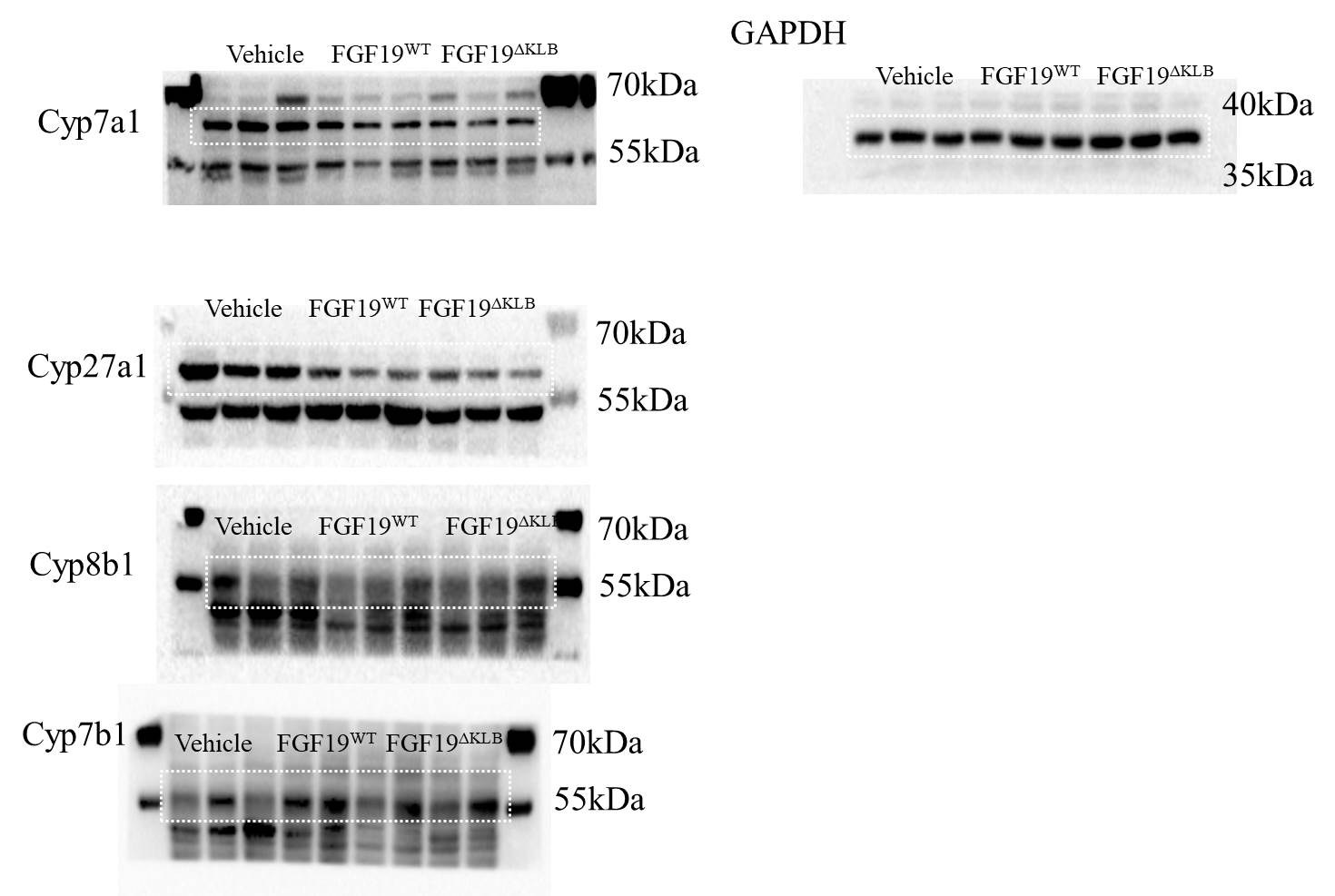


Figure S5.The original blot image of Figure 5C. Western blot analysis of livers from Mdr2-/- mice treated with PBS, FGF19^WT^ or FGF19^ΔKLB^ using primary antibodies against Cyp7a1, Cyp27a, Cyp8b1 and Cyp7b1. All cropped blot image parts in the manuscript are highlighted with white dashed frames on the original blot images.

**
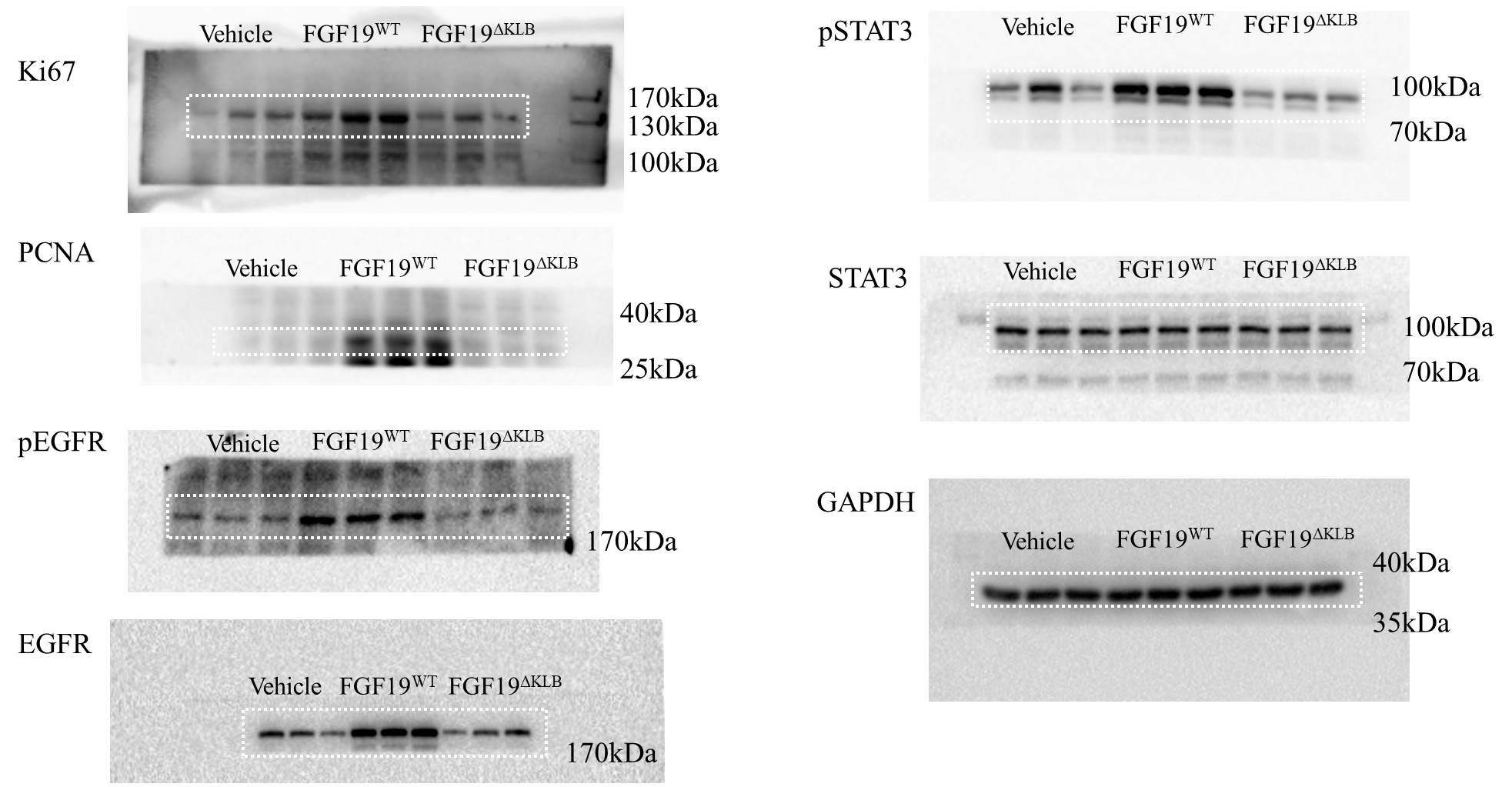
**

Figure S6. The original blot image of Figure 6C. Hepatic protein expressions of Ki67, PCNA, pEGFR, EGFR, pSTAT3 and STAT3 were evaluated by Western blot analysis. All cropped blot image parts in the manuscript are highlighted with white dashed frames on the original blot images.


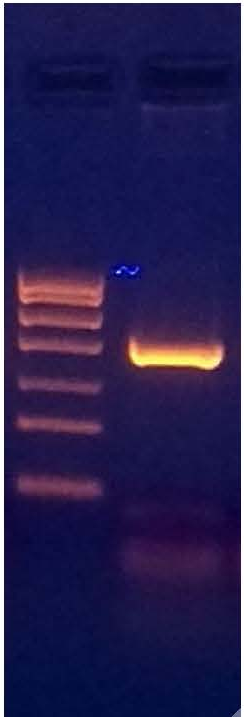


Figure S7.The original blot image of Figure S3C.

Left lane: DNA ladder includes seven DNA markers: 700, 600, 500, 400, 300, 200 and 100 bp from up to down. Right line: Samples of HepG2 cells used in our experiments.

**Supplementary Table 1. Primers of genes used for RT-PCR**

| **Name**  β-actin | **Forward (5’-3’)**  GGCTGTATTCCCCTCCATCG | **Reverse (5’-3’)**  CCAGTTGGTAACAATGCCATGT |
| --- | --- | --- |
| Cyp7a1 | GGGATTGCTGTGGTAGTGAGC | GGTATGGAATCAACCCGTTGTC |
| Cyp8b1 | CTAGGGCCTAAAGGTTCGAGT | GTAGCCGAATAAGCTCAGGAAG |
| Cyp27a1 | CCAGGCACAGGAGAGTACG | GGGCAAGTGCAGCACATAG |
| Cyp7b1 | GGAGCCACGACCCTAGATG | GCCATGCCAAGATAAGGAAGC |
| IL-6  TNFα  TGFβ  col1a1  Col3a1  Bsep  Mrp2 | CTGCAAGAGACTTCCATCCAG  GGCATGGATCTCAAAGACAACC  CCTGCAAGACCATCGACATG  GCTCCTCTTAGGGGCCACT  CTGTAACATGGAAACTGGGGAAA  TCTGACTCAGTGATTCTTCGCA  GTGTGGATTCCCTTGGGCTTT | AGTGGTATAGACAGGTCTGTTGG  AAATCGGCTGACGGTGTGG  GCGAGCCTTAGTTTGGACAG CCACGTCTCACCATTGGGG  CCATAGCTGAACTGAAAACCACC  CCCATAAACATCAGCCAGTTGT  CACAACGAACACCTGCTTGG |
| NTCP | CAAACCTCAGAAGGACCAAACA | GTAGGAGGATTATTCCCGTTGTG |
| OATP1 | GTGCATACCTAGCCAAATCACT | CCAGGCCCATAACCACACATC |
| OATP2 | GGGAACATGCTTCGTGGGATA | GGAGTTATGCGGACACTTCTC |
